# Examination of the psychometric properties of the persian version of the COVID-19-impact on Quality of Life Scale

**DOI:** 10.1186/s12955-021-01829-2

**Published:** 2021-07-30

**Authors:** Ali Hasanpour Dehkordi, Marzieh Aslani, Abbas Ebadi, Selman Repišti, Borhan Moradveisi, Reza Ghanei Gheshlagh

**Affiliations:** 1 Social Determinants of Health Research Center, School of Allied Medical Sciences, Shahrekord University of Medical Sciences, Shahrekord, Iran; 2 Department of Nursing, Asadabad School of Medical Sciences, Asad Abad, Iran; 3 Behavioral Sciences Research Center, Life Style Institute, Baqiyatallah University of Medical Sciences, Tehran, Iran; 4 Psychiatric Clinic, Clinical Centre of Montenegro, Podgorica, Montenegro; 5 Cancer and Immunology Research Center, Research Institute for Health Development, Kurdistan University of Medical Sciences, Sanandaj, Iran; 6 Spiritual Health Research Center, Research Institute for Health Development, Kurdistan University of Medical Sciences, Sanandaj, Iran

**Keywords:** Psychometric properties, QOL, Covid-19

## Abstract

**Introduction:**

As a result of high transmission and mortality rates, the Covid-19 pandemic has led to a worldwide health crisis, isolation, and widespread fear, therefore negatively influencing people’s quality of life (QOL). The goal of the present study was to examine the psychometric properties (validity and reliability) of the Persian version of the COVID-19-Impact on Quality of Life (COV19-QoL) scale.

**Methods:**

After translating the scale using the forward–backward method, face and content validly was qualitatively assessed. Then the scale was distributed to 488 individuals from the general population via online platforms. Construct validity was assessed using exploratory (EFA) and confirmatory (CFA) factor analysis. In addition, internal consistency was examined using Cronbach’s alpha coefficient and McDonald’s omega, relative stability was assessed using interclass correlation coefficient (ICC), and absolute stability was calculated through examination of standard error of measurement.

**Results:**

The EFA revealed one factor that explained 55.96% of the total variance of the scale. Internal consistencies of 0.823 and 0.882 were found using Cronbach’s alpha coefficient and McDonald’s omega, respectively. In addition, an ICC of 0.837 (with a two-week interval) was found. Covid-19 had a greater impact on the QOL of healthy participants than that of those with underling conditions (*p* = 0.004), and also on the QOL of single participants than that of married ones (*p* = 0.032).

**Conclusion:**

The Persian version of the COV19-QoL is a valid and reliable instrument that can be used to examine the impact of Covid-19 on QOL.

## Introduction

On December 2019, the outbreak of an unknown pneumonia was reported in Wuhan, China (1). Shortly after, the new coronavirus (SARS-CoV-2) was confirmed as the cause of the pandemic in China and many other parts of the world (2). On January 30, 2020, the world health organization (WHO) declared the pandemic a public health emergency (3). On March 24, 2020, the number of COVID-19 confirmed cases and deaths were 372,755 and 16,231, respectively; by April 18, 2020, these increased to 2,160,207 and 146,088, respectively (4).

The fast prevalence of COVID-19 and the high death rates has led to widespread anxiety (5), fear and panic (6), psychological distress (7), depression (8), Post-traumatic stress symptoms (9), insomnia (10) and even stigma and xenophobia towards suspected cases (11). Many of these fears are related to getting the virus, losing loved ones, lack of medical treatment, and issues related to staying at home, unemployment, and job loss that lead to psychological disorders, and in some cases suicide (12, 13). A study conducted in India found a significant association between hours spent on watching news on COVID-19 and level of distress and anxiety experienced (14). In addition, false information about COVID-19, travel ban, and quarantine all can affect people’s psychological health (15). Therefore, governments should focus on disseminating unbiased COVID-19 knowledge, teaching the correct methods of restraint, providing basic services and goods, and adequate financial support (16).

The pandemic has led to closure of schools and jobs, financial vulnerability, and loss of many jobs (17), and has created many problems for people around the world. The results of various studies have shown that lockdown and social distancing have led to psychological distress in people (especially women, the elderly and people with many children) and had adverse effects on economic well-being and quality of life (18, 19). Some precautionary measures, such as compulsory face mask policies, depended on communities' previous experience with epidemics. For instance, due to the previous experience of the Chinese people in the SARS epidemic in 2003, the use of masks in the Chinese people was more than in Poland, while the use of masks in Poland was considered stigma (20).

Different aspects of daily life and people’s quality of life (QOL) have been affected by the rapid spread of the virus. QOL is a subjective, multidimensional, and personal concept that is defined as one’s perception of their life, values, objectives, standards, and interests, and refers to one’s attitude towards the difference between what is and what ought to be (21, 22). As a result of unprecedented death rates, unemployment, and isolation, life has become harder for many people during the pandemic (23). It is clear that from now on the world has to live with this virus; therefore, QOL and other psychological and demographic issues related to it should be examined, so that proper measures can be taken. Some studies have examined QOL using general instruments, such as the SF-36 (24, 25) and the SF-12 (26). Some other studies have used instruments measuring psychological problems and social support that do not seem to properly assess QOL (2, 27). Therefore, it seems necessary to have a scale specifically assessing QOL. The COVID-19–Impact on Quality of Life (COV19-QoL) scale is a standard and specific scale for assessing QOL during the COVID-19 pandemic that was developed by Repišti et al. It has good validity and reliability, and assesses the main dimensions of QOL in the general population (28). The goal of the present study is to examine the psychometric properties (validity and reliability) of the Persian version of the COV19-QoL.

## Methods

### Study design and environment

This is a methodological, cross-sectional study aimed at examining the psychometric properties (validity and reliability) of the COV19-QoL in the Iranian general population in 2020. The scale was distributed to the participants via online platforms.

### Sample size and participants

The minimum sample size required to perform exploratory factor analysis is 3 to 10 samples per item. There is debate on the proper sample size for EFA. Some researchers suggest that 5–10 participants are needed per item. However, sample sizes of 150–300 and even 300–500 participants have also been suggested (29). In conducting a confirmatory factors analysis (CFA), sample size should not be lower than 200 (30). In the present study, the sample included 488 participants who were randomly divided into two groups, a 288-memebr group for the EFA and a 200-memebr group for the CFA.

### Procedure

Participants were recruited in the study using anonymous online survey and snowball sampling method. Brief information about the study and a webpage link to the study, were shared via WhatsApp and Telegram. The online survey was administered by Porsline (which is equivalent to Google form). Distributing the questionnaire link through various channels on social networks in Iran is a type of advertising and requires payment. Therefore, the link was distributed in several free scientific channels and participants were asked to share this link with others. After agreeing to complete the survey, participants completed demographic information and COV19-QoL questionnaire. Answering all the questionnaire items were required and the respondents could only submit the form if they answered all the questions.

### Instruments

The COV19-QoL assesses the respondent’s QOL in the past week during the COVID-19 pandemic. It has 6 items rated on a 5-point Likert-type scale ranging from 1 (totally agree) to 5 (totally disagree). Total score is the sum of item scores, and higher scores indicate a more severe impact of COVID-19 on QOL (28). The COV19-QoL was administered to 1346 participants from Croatian general population (non-clinical sample) and 201 patients with severe mental illness recruited from Bosnia and Herzegovina, Montenegro, North Macedonia and Serbia (clinical sample). Item #1 assesses the respondent’s feelings about the effects of COVID-19 on their overall QOL. Items #2 and #3 assess the respondent’s perception of possible deterioration in their mental and physical health. Items #4 and #5 assess anxiety and depression related to physical health and risk of being contaminated. The last item assesses the respondent’s perception of personal safety (28).

### Translation process

First of all, the approval of the developer of the scale was obtained to translate it into Persian. Then, the scale was translated using the forward and backward method, so that it was first translated from English to Persian, and then back-translated into English by two other translators. The final version of the scale was developed by the research team (31). In order to increase the accuracy of the study, the cooperation of the original developer of the scale was sought.

### Face and content validity

Face and content validity were assessed qualitatively. Cognitive interviews were used to assess face validity. In addition, 10 experts reviewed the items, and determined ambiguous items. Content validity shows the degree an instrument covers the concepts of interest (32). In order to assess content validity, 5 experts (two nurses, a health promotion expert, a psychiatrist, and a sociologist) were asked to assess the content of the Persian version of the scale. The ceiling and floor effects were also examined. When more than 15% of respondents obtain the lowest or highest possible scores, floor or ceiling effect is present, respectively (33), and the presence of these effects indicates that extreme items may be missing in the upper or lower end of the scale; this shows inadequate content validity (34).

### Data analysis

The data was analyzed using PASW v18 and LISREL v8.8, as follows:

### Construct validity

An EFA using Maximum likelihood and Promax rotation was used to assess underlying (or latent) relationships between the variables. Sampling adequacy was examined using Kaiser–Meyer–Olkin (KMO) coefficient. KMO values ranging from 0.7 to 0.8 and from 0.8 to 0.9 are considered good and great, respectively. High KMO values (more than 0.7) usually show that factor analysis is appropriate for the data (35). The Bartlett’s test of sphericity was used to examine the significance of the correlation matrix between the variables. A cut-off point of 0.30 was considered for factor loadings. The CFA was performed to examine whether the data conform to the theoretical model. It was conducted based on the results of the EFA using maximum likelihood estimation method and covariance indices. Goodness-of-fit was assessed using goodness-of-fit index (GFI), relative chi-square (χ2/df), normed fit index (NFI), comparative fit index (CFI), standardized root mean square residual (SRMR), and root mean square error of approximation (RMSEA) (36). The following values were considered acceptable for the aforementioned indices: χ2/df ≤ 2, GFI, CFI, and NFI > 0.95, RMSEA < 0.06, and SRMR < 0.08 (37, 38). Independent t-test and one-way analysis of variance (ANOVA) were used to compare the mean scores of quality of life in the two groups and more than the two groups, respectively.

### Reliability

Internal consistency was assessed using Cronbach’s alpha coefficient and McDonald's omega, and relative stability was examined using interclass correlation coefficient (ICC) with two-way mixed effects, and 95% confidence interval; ICC values higher than 0.75 are regarded acceptable (39). Absolute stability was calculated by assessing standard error of measurement (SEM).

SEM was calculated using the following formula: SEM = SDbaseline × √(1 − ICC), and MDC was calculated by the following formula: MDC = 1.96 × √(2) × SEM. MDC is defined as the minimal amount of change that is not likely to be a result of measurement error (40). According to the classical test theory, error is the difference between a true score and an observed score (41, 42). Therefore, when measuring an individual, the true score may not be known as a result of variation in measurement. The threshold provided by the MDC data allows researchers to be confident that above a certain point, changes in scores are not just due to measurement error (43).

### Ethical considerations

The present study is based on a research project approved by the research committee at Shahrekord University of Medical Sciences (IR.SKUMS.REC.1399.202). Before starting the study, the objectives were explained to the participants. In addition, the participants were not required to write down their real names on the questionnaires. Moreover, they were reassured that their personal information remained confidential.

## Results

The sample included 488 individuals with a mean age of 28.90 ± 11.51 years. The majority of participants were female (75.6%), single (54.3%) and with college education (66.2%). Table 1 presents the average quality of life score by demographic characteristics. The impact of COVID-19 was higher on healthy participants than those with underlying conditions (16.81 ± 5.67 vs. 14.75 ± 5.68, *p* = 0.004), and also on single participants than those who were married (16.97 ± 5.51 vs. 15.86 ± 5.90, *p* = 0.032). In addition, there was no association between QOL and age, gender and education. The mean score of each item by demographic variables is presented in Table 2. The mean score of all items was higher in men than women, in single people more than married people and in healthy people more than people with comorbidities. Gender group showed a significant difference only for item I2, with males having higher scores. Marital status revealed a significant difference only for I3 and I6 items; conversely, educational status showed a significant difference for all items except for I2 and I6. Educational status of the population did not reveal any significant difference in the mean item score for any items. Total score on the COV19-QoL is calculated by summing the scores of the six items, and higher scores indicate better QOL. The mean QOL score was 16.46 ± 5.71.

**Table 1:** Mean score of quality of life by demographic variables

Variable	n	%	Mean (SD)	Statistical test	Results
**Gender**					
Male	119	24.4	17.13 (6.08)	Independent T test	*p* = 0.145 t = 1.461
Female	369	75.6	16.25 (5.58)		
**Marital status**					
Married	265	54.3	15.86 (5.90)	Independent T test	0.032 t = 2.148
Single	223	45.7	16.97 (5.51)		
**Comorbidity**					
Yes	81	16.6	14.75 (5.68)	Independent T test	*p* = 0.030 t = 2.981
No	407	83.4	16.81 (5.67)		
**Educational Level**					
Elementary	42	8.6	15.90 (7.45)	One-way analysis of variance	0.213 F = 1.461
High school/ Diploma	123	25.2	16.93 (5.66)		
University degree	323	66.2	16.36 (5.48)

**Table 2:** Item wise mean score comparison based on demographic variables

Variable	I1	I2	I3	I4	I5	I6
**Gender**						
Male	2.44 (1.23)	2.95 (1.29)	3.07 (1.21)	2.62 (1.14)	2.95 (1.36)	3.07 (1.30)
Female	2.38 (1.13)	2.64 (1.14)	3.05 (1.20)	2.49 (1.10)	2.70 (1.20)	2.97 (1.26)
*p* value^†^	0.637	0.013^*^	0.866	0.284	0.293	0.434
**Marital**						
Single	2.45 (1.12)	2.81 (1.15)	3.16 (1.15)	2.56 (1.10)	2.84 (1.28)	3.13 (1.26)
Married	2.34 (1.20)	2.60 (1.23)	2.92 (1.25)	2.48 (1.11)	2.66 (1.20)	2.83 (1.27)
*p* value^†^	0.289	0.058	0.029^*^	0.442	0.119	0.010^*^
**Comorbidity**						
No	2.45 (1.16)	2.75 (1.17)	3.15 (1.19)	2.57 (1.09)	2.83 (1.23)	3.02 (1.27)
Yes	2.12 (1.09)	2.53 (1.29)	2.59 (1.15)	2.25 (1.17)	2.41 (1.25)	2.82 (1.30)
*p* value^†^	0.018^*^	0.116	0.001^*^	0.018^*^	0.006^*^	0.193
**Educational status**						
Elementary	2.40 (1.36)	2.57 (1.36)	2.69 (1.37)	2.66 (1.35)	2.66 (1.42)	2.90 (1.55)
High school/ Diploma	2.50 (1.14)	2.73 (1.16)	3.09 (1.21)	2.69 (1.12)	2.88 (1.24)	3.00 (1.25)
University degree	2.36 (1.14)	2.73 (1.18)	3.09 (1.17)	2.44 (1.06)	2.73 (1.22)	3.00 (1.24)
**P value**^**††**^	0.516	0.696	0.117	0.065	0.437	0.889

^*^p < 0.05

^†^Based on independent samples *t* test

^††^Based on one-way ANOVA

Due to the simplicity of the items and the accurate translation, which was done under the supervision of the original designer, none of the items changed in face and content validity. In addition, both the ceiling and floor effects were found to be 0 and 2%, respectively, indicating good content validity of the scale.

## Construct validity

### Exploratory factor analysis (EFA)

A KMO value of 0.894 was found, and Bartlett’s test of sphericity was significant (*X*^*2*^ = 1400.651, *df* = 15, p = 0.001). The analysis revealed one factor that explained 55.967% of the total variance. (Table 3).

**Table 3:** Exploratory factor analysis of the Persian version of the COV19-QoL

Due to the COVID-19 pandemic	Factor loading	Mean (SD)	h^2^
1. …I think I have a lower quality of life than before	0.691	2.40 (1.16)	0.477
2. …I believe my mental health has declined	0.814	2.72 (1.19)	0.662
3. …I think my physical health may decline	0.708	3.05 (1.20)	0.502
4. …I am more tense than before	0.754	2.52 (1.11)	0.569
5. …I am more depressed than before	0.814	2.76 (1.24)	0.663
6. …I feel that my safety is in danger	0.697	2.99 (1.27)	0.486
% variance	55.967		
Eigen value	3.789		
Internal consistency	Omega = 0.882; Alpha = 0.823		

M: Mean; SD: Standard deviation

### Confirmatory factor analysis

**Fig. 1:**
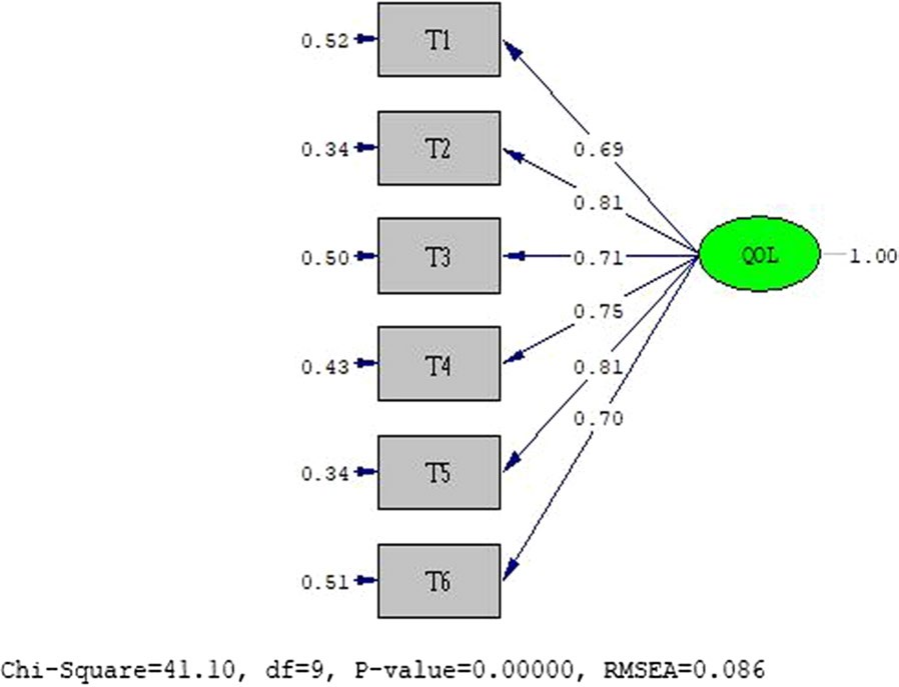
The final model

In the CFA, the model had a good fit. The examined goodness fit indices were as follows: normal fit index (NFI) = 0.98, root mean square error of approximation (RMSEA) = 0.052, goodness-of-fit index (GFI) = 0.0.97, standardized root mean square residual (SRMR) = 0.030, comparative fit index (CFI) = 0.963, and incremental fit index (IFI) = 0.99. The results of the CFA are presented in Fig. 1.

### Reliability

Internal consistency of the scale was found to be 0.823 and 0.882 using Cronbach’s alpha coefficient and McDonald’s omega, respectively. In addition, relative stability of the scale with a two-week interval was found to be 0.837 (95% confidence interval: 0.686–0.935). Examination of absolute stability revealed a SEM of 1.8 and a MDC of 3.5.

## Discussion

Validity and reliability are important indicators of instruments. The former indicates the accuracy of an instrument and the latter shows its stability (44). The present study was aimed at assessing the validity and reliability of the Persian version of the COV19-QoL. This tool has been designed to assess the effects of COVID-19 on QOL of people from the general population. The fast spread of COVID-19 has led to significant stress and anxiety among populations, and due to isolation, closure of schools, and loss of jobs, people may experience high levels of distress and their QOL of may be negatively influenced; therefore, assessing the effects of pandemic on QOL has an important role in taking necessary measures to combat these problems.

Under the supervision of the original tool designer, the translation process was carried out so the final Iranian version accurately represents the original one. Due to the small number of items and carefully guided translation, nothing changed in its face validity and content. Ceiling and floor effects were found to be 0% and 2% respectively, indicating that items showing the maximum and minimum intensity of the phenomenon are included in the scale, and that it has good content validity (33).

The COV19-QoL is a unidimensional instrument assessing QOL during the COVID-19 pandemic. According to the results of EFA, the variance explained for each item was higher than 0.4, and all factor loadings were higher than 0.6; these results were consistent with those found for the original version of the scale (28). In the original version of the scale, the highest factor loadings in the clinical (0.861) and non-clinical (878) samples were for item #4 (I am more tense than before). In the Persian version, the highest factor loadings were for items #5 (I am more depressed than before) and #2 (I think my mental health has declined) that both had a factor loading of 0.814. This difference can be attributed to cultural differences between the populations under study and different effects of COVID-19 on their QOL.

Internal consistency of the original version of the scale, based on Cronbach’s alpha coefficient, was found to be 0.856 and 0.885 in clinical and non-clinical samples, respectively (28). In addition, internal consistency of the Persian version of the scale was found to be 0.882 and 0.823 using Cronbach’s alpha coefficient and McDonald's omega. In contrast to the original version, absolute stability of the Persian version using SEM and MDS was found to be 1.8 and 3.5, respectively. SEM = 3.5 shows that if there is 3.5 points change in the total score after the intervention, we can be 95% confident that a true change has been occurred in QOL. The COV19-QoL had acceptable stability and internal consistency. Although a low Cronbach’s alpha coefficient was expected due to low number of items, an alpha of 0.8 was found that was great.

The results showed that the COV19-QoL scores were higher in healthy individuals than those with underlying conditions, and also in single than married participants. Healthy individuals may be more concerned about their health than those with underlying conditions. In other words, people with underlying conditions are more able to adjust to the COVID-19 pandemic, because they have experienced other conditions. In the original version, the quality of life of healthy people (non-clinical samples) was more affected compared to patients (clinical sample) and the mean score of all items of COV-19QoL in healthy people was higher than patients (28).

In addition, single individuals may experience more problems during the pandemic due to receiving less social support. A review study by Sanyaolu et al. showed that people with underlying conditions, such as diabetes and high blood pressure were more likely to die from COVID-19 than healthy individuals (45). Samlani et al. examined the QOL of their participants during the COVID-19 pandemic using the SF-12. The results of this study showed that there was a higher decrease in the QOL of participants compared to the quality of their physical life (26). In a study among cancer patients, Greco et al. found a higher decrease in the psychological aspect of QOL than its physical aspect during the COVID-19 pandemic (25).

Two main advantages of the present study were identified. They include (1) the use of CFA which was not conducted in the original study on the scale validity and (2) the calculation of two coefficients to examine the reliability of the scale. The main limitation of the study, as in most correlational studies, was the snowball (that is, non-probabilistic) sampling which does not guarantee that the findings and conclusions can be generalized without reasonable caution. Another limitation of this study was the lack of convergent validity.

## Conclusion

The COV19-QoL is a valid and reliable instrument to assess the effects of COVID-19 on the quality of life of the general population. Therefore, it can be used in future studies.

## Data Availability

The datasets used and/or analyzed during the current study are available from the corresponding author on reasonable request.

## Abbreviations

CFA: Confirmatory factor analysis
CFI: Comparative fit index
COV19-QoL: COVID-19-impact on Quality of Life
EFA: Exploratory factor analysis
GFI: Goodness-of-fit index
ICC: Interclass correlation coefficient
KMO: Kaiser–Meyer–Olkin
MDC: Minimal detectable change
NFI: Normed fit index
PASW: Predictive Analytics SoftWare
QoL: Quality of life
RMSEA: Root mean square error of approximation (RMSEA)
SEM: Standard error of measurement
SRMR: Standardized root mean square residual
WHO: World Health Organization
